# Identification of Novel Retroid Agents in *Danio rerio*, *Oryzias latipes*, *Gasterosteus aculeatus* and *Tetraodon nigroviridis*

**Published:** 2007-09-06

**Authors:** Holly A. Basta, Alex J. Buzak, Marcella A. McClure

**Affiliations:** Department of Microbiology and the Center for Computational Biology, Montana State University at Bozeman, 109 Lewis Hall, Bozeman, MT 59717, U.S.A.

**Keywords:** Retroid, transposable elements, Genome Parsing Suite software, Danio rerio, Oryzias latipes, Gasterosteus aculeatus, Tetraodon nigroviridis

## Abstract

Retroid agents are genomes that encode a reverse transcriptase (RT) and replicate or transpose by way of an RNA intermediate. The Genome Parsing Suite (GPS) is software created to identify and characterize Retroid agents in any genome database (McClure et al. 2005). The detailed analysis of all Retroid agents found by the GPS in *Danio rerio* (zebrafish*), Oryzias latipes* (medaka)*, Gasterosteus aculeatus* (stickleback) and *Tetraodon nigroviridis* (spotted green pufferfish) reveals extensive Retroid agent diversity in the compact genomes of all four fish. Novel Retroid agents were identified by the GPS software: the telomerase reverse transcriptase (TERT) in *O. latipes*, *G. aculeatus* and *T. nigroviridis* and a potential TERT in *D. rerio*, a retrotransposon in *D. rerio*, and multiple lineages of endogenous retroviruses (ERVs) in *D. rerio*, *O. latipes* and *G. aculeatus*.

## Introduction

Retroid agents are genomes that use the RT to transcribe their RNA into dsDNA, which is then integrated into the DNA of a host organism. Once falsely labeled “junk” DNA, some Retroid agents are implicated in disease, while others are beneficial to the organism in which they reside. In humans the HERV W envelope protein is essential for placental reproduction (Blond et al. 2000, Mi et al. 2000, Mallet et al. 2004), while other Retroid agents provide regulatory sequences for the host genes (Samuelson et al. 1988; Yang et al. 1998; Medstrand et al. 2001), maintain telomeres (Pardue et al. 1996), repair damaged chromosomes (Moore et al. 1996), carry genetic information within an organism, and also transport genetic information to other organisms (Kordis et al. 1998).

The Retroid agent classification includes both endogenous and exogenous retroviruses, pararetroviruses (large DNA viruses), retrotransposons with long terminal repeats (LTRs), retroposons that lack LTRs, retroplasmids, retrointrons, and retrons (Temin, 1985; Temin, 1989; Hull, 1999; Hull, 2001). Although the effects of Retroid agents have been studied in far more detail in the mammalian and insect genomes, it is apparent that at least one of these genes, the telomerase reverse transcriptase (TERT), performs a similar function, repairing the telomeric ends in the four teleost fish. In mammals the TERT plays important roles in cell proliferation, differentiation, tumorigenesis and aging. The *Takifugu rubripes* (Japanese pufferfish) TERT (FTERT) is essential for maintaining the ends of linear chromosomes (Yap et al. 2005). The TERT is the only RT function encoded by a host gene; all other cellular RT activity is encoded by Retroid genomes (McClure, 1999).

The four teleost fish, *D. rerio* (zebrafish)*, O. latipes* (medaka), *G. aculeatus* (stickleback) and *T. nigroviridis* (green spotted pufferfish) are of particular interest both experimentally and evolutionarily. *D. rerio* is an important model organism for vertebrate development (Kimmel, 1989; Driever et al. 1996) and organogenesis (Zhong et al. 2000). This freshwater fish is also used to determine the roles of hundreds of essential vertebrate genes. Another freshwater fish, *O. latipes,* is an important model for the evolution of sex determination and developmental genetics (Shima et al. 2003). *G. aculeatus* is a small marine fish which has undergone one of the most rapid and recent adaptive radiations on earth. Sticklebacks normally live in the ocean and migrate into freshwater streams and lakes to breed. During the last Ice Age, however, many ocean sticklebacks colonized newly created lakes and streams, and in many cases became isolated from the ocean (Bell et al. 1994), therefore, the stickleback is a very important asset in the study of evolution. The freshwater pufferfish is a model for genomic studies because it has the smallest genome size of all vertebrates sequenced to date (Shima et al. 2003). The zebrafish genome, about 1700 Mbp, is the largest, followed by the medaka, with a genome of approximately 1000 Mbp, then the stickleback with 675 Mbp, while the pufferfish genome, with 385 Mbp, is the smallest (Volff, 2005). The compact size of *T. nigroviridis* can be attributed to the small number of repetitive agents, in addition to its reduced intron size (Jallion et al. 2004). The divergence times of the four fish follow the same trend as the genome sizes: *D. rerio* is the oldest, followed by *O. latipes*, then *G. aculeatus* while *T. nigroviridis* is the most recently diverged (Yamanoue et al. 2006).

The results reported here are from the GPS software used for identification, classification and comparison of the Retroid agent content of the *D. rerio, O. latipes, G. aculeatus* and *T. nigroviridis* genomes. The approach of the GPS is radically different from Repeat Masker, which is used to mask out and count repetitive agents using consensus DNA sequences (Smit et al. 1996–2004). Other approaches employ LTR sequences to find a subset of Retroid agents (Buzdin et al. 2006). These methods are limited to finding Retroid sequences that can be detected by a library of DNA sequences. These methods suffer from the loss of signal due to mutational saturation because DNA is used to query a genome rather than amino acid sequences. While the structural genes of Retroid agents can be highly divergent, the RT gene is considerably more conserved (McClure et al. 1988). The RT is also essential for autonomous transposition, and the continuance of an exogenous viral life cycle. This being said, in the studies presented here, we have limited the GPS analysis to identify those Retroid agents capable of autonomous replication. Although any protein sequence can be used in the GPS, in this study it is populated with a representative diversity of RT protein sequences which afford a deep query into the Retroid content of the *D. rerio, O. latipes, G. aculeatus* and *T. nigroviridis* genomes.

## Methods

### The genome parsing suite

#### Stage I GPS

Washington University Basic Local Alignment Search Tool translated nucleotides (WU-tBLASTn) version 2.0 (Gish 1996–2004) was used to query the four fish genomes with the following parameters: E = 1, –matrix pam70, Q = 9, R = 1, V= 1e7, B = 1e7, gapL = 0.307, gapK = 0.13, gapH = 0.7, X = 15, gapX = 33, gapW =44, gapS2 = 63, S = 41, hspmax = 0, and –span.

**Figure 1.**
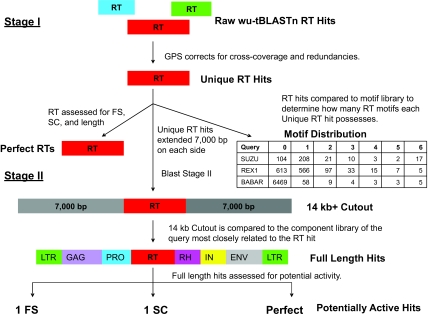
A Representation of the GPS Software. The output of Stage I GPS includes Raw, Unique and Perfect RT hits as defined in the text. Unique hits are assessed for presence of the Ordered Series of Motifs (OSM) as illustrated in the Motif Distribution box. The columns indicate the number of motifs the Unique RT hits have (zero through six), and the rows indicate queries. For example, there are 17 copies of an RT sequence with all six motifs that is more closely related to the retrotransposon SUZU than any other query. All Unique hits are passed to Stage II GPS and a 14kb^+^ length of host DNA inclusive of each RT hit is excised and assessed. The results of this stage are full length Retroid genomes, classified as those with one stop codon (1SC) or frame shift, (1FS) and those with complete, error-free open reading frames (perfect). Given the observation of translational recoding in Retroid agents, these three classes are considered potentially active. Gene abbreviations are as follows: LTR = long terminal repeat, GAG = group specific antigen, PRO=protease, RT = reverse transcriptase, RH = ribonuclease H, IN = integrase, and ENV = envelope.

Figure 1 outlines the two stages of the GPS software. Stage I sorts and filters raw WU-tBLASTn hits retrieved by the RT queries. These hits are redundant and contain false positives, due to: 1) alternative alignments for a given query to a specific region, 2) cross coverage of the queries, and 3) counting as unique, a number of small hits that are actually from the same gene. After sorting by query, chromosome, polarity and reading frame the GPS removes redundancy by deleting hits that are completely covered by a longer hit to the same position, thereby preventing overestimation of the amount of potential RT genes. The GPS then compounds small hits, and removes false positives due to cross coverage on these compounded hits. These filtered data are the “unique” RT hits. Unique hits are counted as single contiguous sequences, single compound hits composed of subsequences, and sets of ambiguous hits to the same position and reading direction. These ambiguous cases are often resolved in Stage II of the GPS. Unique hits are then assessed for quality: 1) by degree of Ordered Series of Motifs (OSM) conservation (McClure, 1991), which is made up of six highly conserved motifs that fold to form the active site of the enzyme (Kohlstaedt et al. 1992), and 2) presence of frame shifts and stop codons (Figure 1). Full length RT hits with neither frame shifts nor stop codons are labeled “perfect”.

#### Stage II GPS

**Figure 2.**
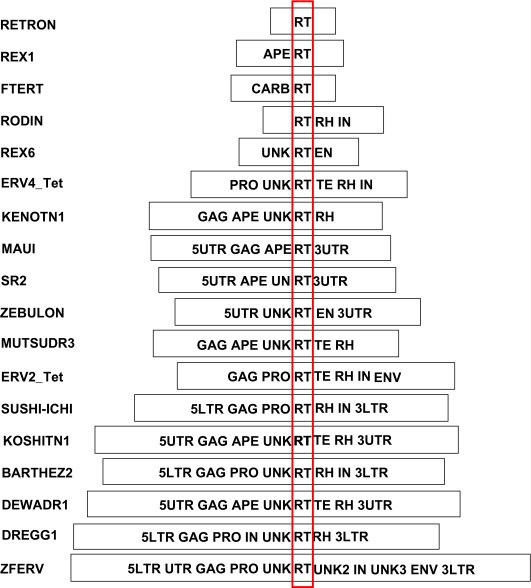
Example Query Library Gene Component Maps. The GPS accesses a database populated by Retroid genomes. This query library contains all the genes and non-coding components, which the GPS uses to identify and reconstruct potential Retroid agents found in organismal genome databases. Those gene abbreviations not found in Figure 1 are as follows: APE = apurinic endonuclease, UN/UNK= unknown region, EN= putative PDD endonuclease (Xiong et al 1988), TE=tether, 5UTR= 5′ untranslated region, 3UTR= 3′ untranslated region, 5LTR= 5′ long terminal repeat, 3LTR= 3′ long terminal repeat. The FTERT is divided into a Carboxyl portion (CARB) and the RT. The red box highlights the RT genes. If a potential Retroid agent encodes all the genes in a specific query component library, it is considered full length. Retroid agents accession numbers and the hosts in which they were discovered are presented in Figure 3.

Stage II GPS uses an RT-outward approach to construct potential Retroid agent genomes. The sequence of each unique RT hit is extended approximately 7,000 bp upstream and downstream of its position in the chromosome (Figure 1, Stage II). Given that the largest size of a Retroid genome is about 9,000 bp, this 14kb^+^ sequence is sufficient to encompass any newly identified Retroid agent. WU-tBLASTn is used a second time to compare each query-specific component library (Figure 2) to the corresponding 14 kb^+^ sequence containing the RT hit. Full length Retroid genomes are defined by the presence of *all* the gene components in the query’s genomic order. Unlike other methods, the GPS does not limit the definition of full length to only sequences bounded by LTRs or untranslated regions (UTRs). It is known that many LTR or UTR bounded Retroid genomes have deletions within these boundaries and, therefore, are not full length. LTRs are analyzed at the amino acid level in the GPS, even though they do not physically encode amino acids, allowing more divergent LTRs to be identified. Note, however, in our study that full length genomes may have only one gene, the RT, while others have many more genes (Figure 2). All genomes with one frame shift or stop codon, as well as those that are error free, are considered to be potentially active. Retroid agents are known to overcome mutational errors of one stop codon or frame shift by translational recoding (for Review, see Baranov et al. 2002) thereby producing functional proteins. Note that some queries themselves may contain frame shifts and stop codons. For a more in depth discussion of the GPS, see McClure et al. 2005.

### Retroid agent queries

**Figure 3.**
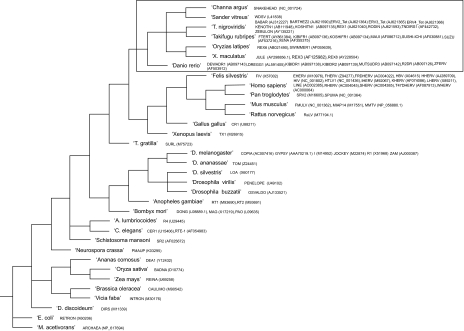
Phylogenetic Tree of the Query Host Organisms. The query names and NCBI accession numbers are listed next to respective host organisms. The tree was created using TaxBrowser on the NCBI website (Benson et al. 2000; Wheeler et al. 2000). Those sequences that are considered fish-specific (see Results) are enclosed in the box.

The phylogenetic tree representing the host organisms of the 92 Retroid agent queries used to populate the GPS in this study, along with the sequence names and accession numbers are presented in Figure 3. The query sequences are: *D. rerio, O. latipes* and *T. nigroviridis* specific; any documented Retroid agents found in any of the three fish genomes; human specific; and a set of 30 that represent the major families of all Retroid agents. There are currently no Retroid agent sequences available from *G. aculeatus*.

### Host genomes

The *D. rerio* version Zv6, the *G. aculeatus* version gasAcu1 and the *T. nigroviridis* tetNig1 chromosomal genomes are from the University of Santa Cruz Genome Bioinformatics Website (DiBiase et al. 2006) and the *D. rerio* version Zv4 genome is from the National Center for Biotechnology Information website (NCBI) (http://www.ncbi.nlm.nih.gov/). The GPS analyzes genomes in chromosomal format, and even though all four fish genomes have unplaced regions of their chromosomes, these regions have been formatted into an “Un” or “NC” chromosome, which separates unplaced contigs using strings of “N”s. Both iterations of the *D. rerio* genome were analyzed by the GPS. The number of unique RT sequences retrieved by the queries has increased from 64,199 in version Zv4 to 102,763 in version Zv6. The full length sequences increased from 556 in Zv4 to 1,116 in Zv6. All data presented here are from the most recent version of the *D. rerio* genome and, to our knowledge, this is the first report of the full Retroid agent content for the Zv6 iteration. The *O. latipes* MEDAKA1 genome is from the Ensembl website (Hubbard et al. 2007) in chromosomal format.

About 73% of the zebrafish genome is sequenced and 95% of this is placed on chromosomes. Of the medaka genome about 40% is sequenced and 75% of this is placed on chromosomes. Of the approximately 68% of the stickleback genome that has been sequenced, about 87% is anchored to chromosomes. Approximately 54% of the 90% completed pufferfish genome has been placed and positioned onto chromosomes. About 3.3% of the genome has been assigned a chromosome, but not its position on that chromosome. The remaining 42.7% has neither chromosomal nor position assignment, and is called an Un_random chromosome.

## Results

Although there are numerous unique RT hits for each fish, only about one hit in 50 is part of a full length sequence (Table 1). There are many sequences that have more components than a unique RT, but are smaller than a full length agent. These sequences can be output by the GPS for in-depth analysis of each agent; however, this level of analysis for all 92 agents is beyond the scope of the genome-wide studies presented here.

### Low frequency data

**Figure 4.**
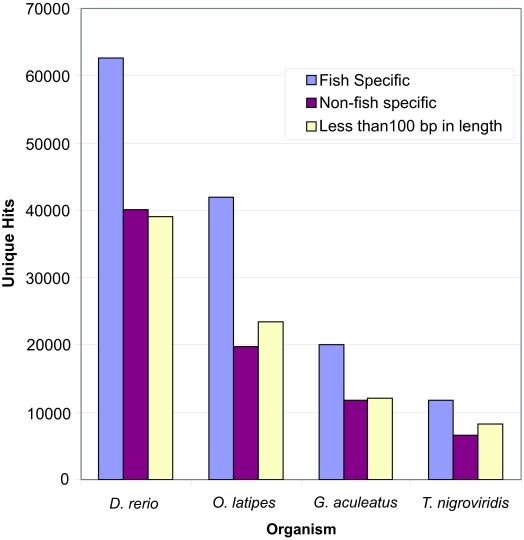
Stage I GPS: low frequency RT hits. Unique RT hits retrieved by fish specific queries are shown in blue. Purple indicates those hits retrieved by non-fish specific queries, while those RT hits that are less than 100 bp in length are shown in off-white. The y axis indicates Unique RT hits, while the x axis indicates organism. See Figure 3 for fish and non-fish specific query names, host organisms and accession numbers.

There are Stage I GPS results that were generated by queries that are not fish specific. This portion of the unique RT copies is referred to as low frequency in previous analyses by the GPS (McClure et al. 2005), and each query finds unique RT copies that are closer to it than they are to any of the other queries. These hits are referred to as low frequency because they make up a small fraction of the human data analyzed in first published report of the GPS (McClure et al. 2005). These unique RT hits do not, however, make up a small portion of the four fish, and will therefore be referred to as non-fish specific hits (Figure 4).

The average size of the RT queries used in this study is about 1050 bp, however, a significant fraction of the total unique RT hits are below 100 bp in all four fish (Figure 4). The majority of these hits under 100 bp belong to non-fish specific sequences. On average, the non-fish specific hits tend to be smaller than those that are fish specific (Table 2). A new function of the GPS will be developed to analyze these fragments of RT sequences, which have been found to some degree in every genome thus far analyzed by the GPS.

### Placement of data onto chromosomes

Almost all of the unique RT sequences found by Stage I GPS have been placed on chromosomes in the *D. rerio* genome, however, the *O. latipes* genome has a large number of non-placed unique RT hits (approximately 25% of the total), as does the *G. aculeatus* genome (approximately 20% of the total) and the *T. nigroviridis* genome (greater than 50% of the total), which is not surprising considering repetitive elements tend to be the most difficult to place on specific chromosomes in the genome assembly process. Given that only 40% of the medaka and 54% of the pufferfish sequenced genomes have been placed and have known positions on chromosomes, little can be concluded about the distribution of Retroid agents in these two fish.

### Types of retroid agents found

**Figure 5.**
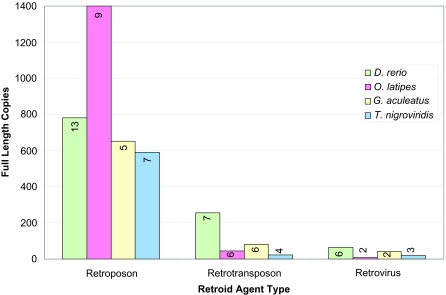
Stage II GPS: number of full length copies by Retroid class and organism. Organisms are represented in different colors: *D. rerio* is light green, *O. latipes* is pink, *G. aculeatus* is off-white and *T. nigroviridis* is light blue. The y axis is the number of full length copies and the x axis is type of agent. Full length copies are separated into retroposons, retrotransposons and retroviruses. Numbers on the bars indicate the number of different retroposon, retrotransposon or retrovirus families that comprise the full length copies indicated by each bar.

All four fish have more unique retroposons than any other type of Retroid agent, followed by retrotransposons and then retroviruses (Figure 5). Predictably, *D. rerio*, with the largest genome of the four fish, has the most unique potential RT genes for all three types of Retroid agents, followed by *O. latipes, G. aculeatus* and then *T. nigroviridis* (data not shown). The full length copies, however, fall into an unexpected pattern according to the sizes of their resident fish; *O. latipes* has more retroposon copies than *D. rerio*, and *G. aculeatus* and *T. nigroviridis* have more retrovirus copies than *O. latipes* (Figure 5). Even though it does not have the most copies of retroposons, *D. rerio* does, however, have more families of each Retroid agent type than any of the other fish (Figure 5).

### Retroid agents present in all four fish

**Figure 6.**
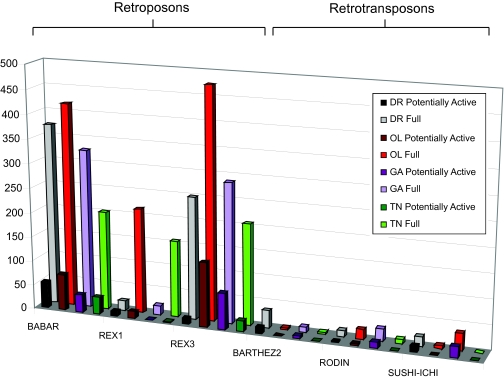
Stage II GPS results for those queries identified as full length in D. rerio (DR), O. latipes (OL), G. aculeatus (GA) and T. nigroviridis (TN). Full length sequences are shown in grey for *D. rerio,* red for *O. latipes,* light purple for *G. aculeatus* and green for *T. nigroviridis.* Full length agents are further classified as potentially active, which includes full lengths with one frame shift, one stop codon, and those agents that have no frame shifts and no stop codons (Perfect). Potentially active sequences are shown in black for *D. rerio,* dark red for *O. latipes,* dark purple for *G. aculeatus* and green for *T. nigroviridis.* The y axis is full length copy numbers and the x axis is the query name. Retroid agents are grouped into retroposon and retrotransposon families. A two-dimensional square indicates zero copies. Their hosts of origin and accession numbers are listed in Figure 3.

There are four types of retroposons and two types of retrotransposons present in all four fish. The copy number trends vary greatly from agent to agent; for example, the REX3 retroposon is most abundant in *O. latipes*, second most in *G. aculeatus*, third most in *D. rerio*, and least in *T. nigroviridis*, while the BARTHEZ2 retrotransposon is most abundant in *D. rerio*, followed by *G. aculeatus, T. nigroviridis* and then *O. latipes* (Figure 6).

### Retroid agents present in three out of the four fish

**Figure 7.**
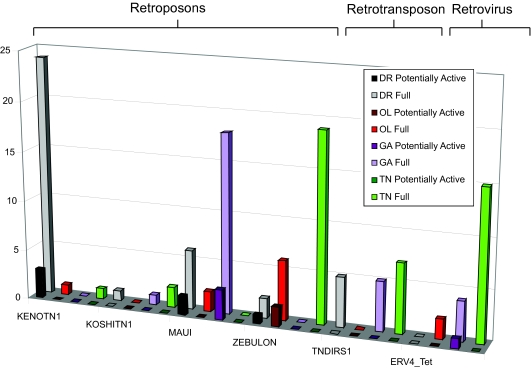
Stage II GPS results for those queries identified as full length in three out of the four fish genomes. Color scheme is as in Figure 6. The y axis is full length copy numbers and the x axis is the query name. Retroid agents are grouped into retroposon, retrotransposon and retrovirus families. A two-dimensional square indicates zero copies. Their hosts of origin and accession numbers are listed in Figure 3.

There are six families of full length Retroid agents found in three out of the four fish (four retroposons, one retrotransposon and one retrovirus). The ERV4_Tet sequences have not been previously identified in either *O. latipes* or *G. aculeatus*, and are currently the focus of a more in-depth study (Figure 7).

### Retroid agents found in two of the four fish and identification of a novel retrotransposon

**Figure 8.**
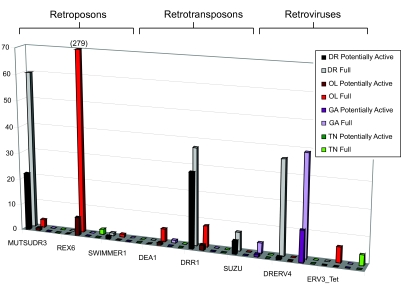
Stage II GPS results for potential full length Retroid agents shared between two of the four fish genomes. Color scheme is as in Figure 6. The y axis is full length copy numbers and the x axis is the query name. Retroid agents are grouped into retroposon, retrotransposon and retrovirus families. Their hosts of origin and accession numbers are listed in Figure 3. Note that the *O. latipes* full length REX6 copies extend beyond the graph, and there are a total of 279 copies. A two-dimensional square indicates zero copies.

There are eight Retroid agent families found in two of the four fish (three retroposons, three retrotransposons and two retroviruses) (Figure 8).

In *D. rerio* there is a genome segment identified as “PREDICTED: *Danio rerio* similar to protease, reverse transcriptase, ribonuclease H, integrase” (NCBI accession number XM_693773). We have further classified this “predicted” agent as a retrotransposon due to its gene content and order, and have identified, in addition to the protease (PRO), RT, ribonuclease H (RH) and integrase (IN), a potential group specific antigen (GAG) region upstream of the PRO. BLAST (Altschul S F, et al. 1990) searches revealed no similarity of the 3′ region to any known envelope (ENV) genes. Neither the 5′ nor the 3′ terminal regions showed any identity to known LTRs upon BLAST analysis through the NCBI (http://www.ncbi.nlm.nih.gov/) viral and non-redundant databases independent of the GPS. We classify this Retroid agent as *D. rerio* retrotransposon 1 (DRR1). These DRR1 copies were initially pulled out by the GPS with the DEA1 query, which is composed of a RT, RH and IN. These sequences warranted closer analysis because the DEA1 query was isolated from *Ananas comosus* (pineapple), and were therefore not expected in fish genomes. These DRR1 sequences were found to be more closely related to each other (about 68% nucleotide identity for the RT), but more distant from the DEA1 query (the DEA1 and DRR1 queries have about 45% amino acid RT identity). These sequences which were initially retrieved by DEA1 are designated as novel retrotransposons. A representative DRR1 query was created using these sequences. Additional components were identified by motifs and BLAST searches. When the 1000 bp upstream and downstream are added to the DRR1 query, an average of about 500 bp both 5′ and 3′ shared high percentage identity: the regions 5′ share an 80.17% nucleotide identity and the regions 3′ share a 75.52% nucleotide identity. When these regions are compared to each other, they have a 69.34% nucleotide identity. Upon integration into a genome, the LTRs are 100% identical, but because only small regions are necessary for regulation they tend to degrade more quickly relative to Retroid genes that encode proteins. The high percentage of conservation found at the 5′ and 3′ termini of the DRR1s suggests that these regions are LTRs. Of the 37 DRR1 sequences that share all components with the query (which did not include the LTRs), 14 of these have potential 5′ and 3′ LTRs. *O. latipes* has eight copies of DRR1 that do not show any identity to either the 5′ or 3′ LTRs of the query, nor do its own 5′ and 3′ regions appear to be related. Both *D. rerio* and *O. latipes* have copies that contain all components of the query, although this query does not contain LTRs, with zero or one frame shift or stop codon (Figure 8).

There are copies of the ERV3_Tet retrovirus in both *O. latipes* and *T. nigroviridis*. The presence of these *O. latipes* retroviruses has been, to our knowledge, previously undocumented, and there are more copies of ERV3_Tet in *O. latipes* than in *T. nigroviridis*. There are no potentially active copies in either fish (Figure 8).

### Retroid agents found in only one of the four fish

Some queries are specific to only one of the four fish (Table 3). Those specific to *D. rerio* include the retroposons: DEWADR1, KIBIDR1, KIBIDR2, and R2DR; the retrotransposon: DREGG1; and the endogenous retrovirus ZFERV. All of these Retroid genomes except KIBIDR1 and ZFERV have potentially active copies. There is only one *O. latipes* specific Retroid agent, the retrotransposon REX8, which is not present in any potentially active copies. The endogenous retrovirus ERV2_Tet is the only *T. nigroviridis* specific Retroid agent, and there are no potentially active copies (Table 3). There are no Retroid agents that are found solely in *G. aculeatus*.

### Identification of novel fish TERTs

**Figure 9.**
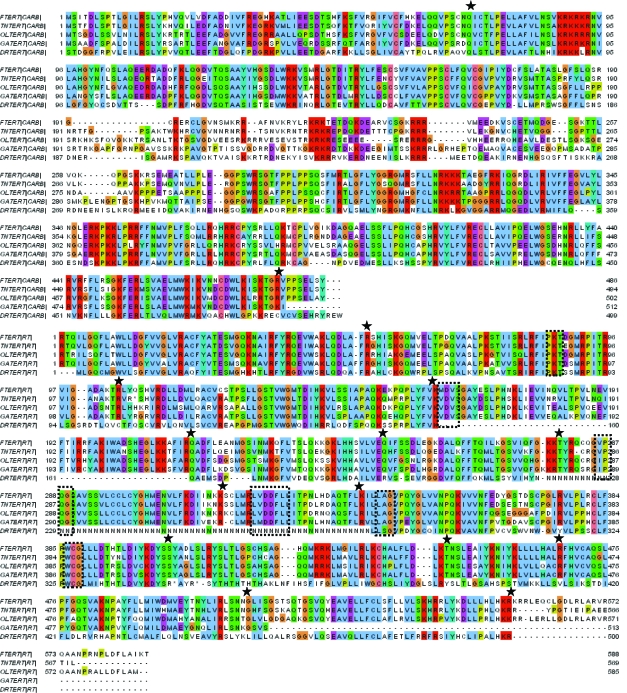
Alignment of the two sections (CARB and RT) of FTERT with the potential TERT proteins found in *T. nigroviridis* (TNTERT), *O. latipes* (OLTERT), *G. aculeatus* (GATERT) and *D. rerio* (DRTERT). The TERT sequences are actually a single long RT, but the sequence is divided into two to increase the chances of finding the entire TERT through multiple introns, as well as to keep it uniform with the rest of the query RT sizes. Note the string of N’s in the *D. rerio* sequence, which is caused by large regions that contain unsequenced regions, annotated by “N” amino acids in the chromosome sequence. Large regions of unsequenced data correspond to only a small portion of the DRTERT because they primarily make up introns that are spliced out when the mRNA is made. This unsequenced portion falls over the second, third, and forth RT motifs. The OSM (see Methods) is indicated by boxes, and the splice points are indicated by stars. The alignment was created using Clustalx in the MEGA 3.0 software package (Kumar et al. 2004).

The previously identified FTERT from *T. rubripes* was used as a query to search for the TERT gene in the fish genomes. We have identified full length TERTs with multiple exons and introns in each of the four fish (Figure 9). Although the TERT functions in fish (Fischer et al. 2000; Kishi et al. 2003; Yu et al. 2006), this is the first published identification of the TERT sequences for these fish. We term these sequences DRTERT (*D. rerio* telomerase reverse transcriptase), OLTERT (*O. latipes* telomerase reverse transcriptase), GATERT (*G. aculeatus* telomerase reverse transcriptase) and TNTERT (*T. nigroviridis* telomerase reverse transcriptase). These TERTs are divided into CARB and RT portions because the four TERTs identified range from 937 to 1087 amino acids, which is far longer than most RT sequences. The TERT sequences are also divided into two regions to help increase the chance of identifying novel TERTs through multiple exons. There are various strings of “N” amino acids where sequencing is incomplete in the regions over which the DRTERT spans, and one of these unsequenced portions falls where the three most conserved RT motifs are expected. These unsequenced portions cause the DRTERT to be about 87% as long as the FTERT query, and make a definitive classification of this sequence difficult before sequencing is complete. Due to the fact that TERTs splice out introns to make mRNAs, these large unsequenced portions only minimally effect the identification of the DRTERT. The first large gap in the RT corresponds to 105 amino acids and a single exon, while the long string of “N” amino acids corresponds to a 234 amino acid region, encompassing two exons. The locations of spliced out introns are indicated by stars in Figure 9. This sequence was also recently submitted to GenBank by Xie, M., Mosig, A., Qi, X., Li, Y., Stadler, P.F. and Chen, J.L. under the accession number EF202140, despite the fact that it is not completely sequenced. This submission was not accompanied by a publication, so the method by which the DRTERT was found is unclear, but alignments confirm that it is indeed the same sequence that the GPS pulls out from the *D. rerio* genome. The four novel fish TERTs are not found on the same chromosome in their respective fish, but all their percent identities are above 40% on the amino acid level (Table 4).

### Identification of novel fish endogenous retroviruses

**Figure 10.**
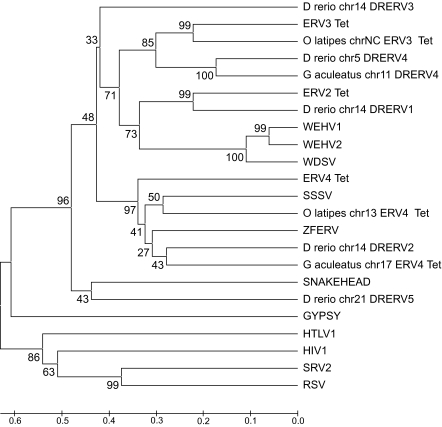
Phylogenetic tree of fish retroviruses. The tree was constructed using RT amino acid sequences from 23 retroviruses (both endogenous and exogenous) and only includes RTs which have all six motifs of the OSM (see Methods). This tree was made using the MEGA 3.0 software (Kumar et al. 2004), using the UPGMA (Sneath et al. 1973) method with bootstrap values (3000 repetitions) (Felsenstein et al. 1985). The tree is drawn to scale, with branch lengths in the same units as those of the evolutionary distances used to infer the phylogenetic tree. The evolutionary distances were computed using the Poisson correction method (Zuckerkandl et al. 1985) and are in the units of the number of amino acid substitutions per site. Organism name, chromosome number, and then Retroid name label tree tips for those novel agents pulled out by a query. The retroviruses and accession numbers that are not included on the query host organism tree (Figure 3) are Walleye epidermal hyperplasia virus type 1 (WEHV1) (AF014792), Walleye epidermal hyperplasia virus type 2 (WEHV1) (AF014793), Atlantic salmon swim bladder sarcoma virus (SSSV) (DQ174103) and Rous sarcoma virus (RSV) (NC_001407). In addition to RSV, GYPSY, HTLV1, HIV1 and SRV2 (Figure 3) are included as non-fish-retrovirus out groups.

When the *D. rerio* genome was originally analyzed for Retroid content by the GPS, the *T. nigroviridis* ERV2_Tet query identified eleven copies and the ERV3_Tet query identified twelve copies of *D. rerio* ERVs. Upon literature and database searches, these agents appear to be previously unidentified, and we have named them DRERVs (*D. rerio* endogenous retroviruses). The RT protein sequence of the DRERV copies pulled out of the *D. rerio* genome using ERV_Tet sequences as queries were used to construct a phylogenetic tree (data not shown). This tree indicated that there are five distinct clades of *D. rerio* ERVs. A representative sequence was chosen from each clade to create five DRERV queries. When these queries were used in the GPS, they pulled out 61 DRERVs; 38 more than the ERV_Tet queries retrieved. Some DRERVs possess more viral gene components than do the ERV_Tet genomes that originally identified these new viruses, and fall into clades distinct from the ERV_Tet clades (Figure 10). The additional components were identified by their motifs in combination with BLAST searches. Five DRERV queries identify 61 full length copies and nine potentially active DRERVs in *D. rerio* (Table 3a). The full length DRERV1 copies have an average RT amino acid identity of 88%, DRERV3 copies have 84%, DRERV4 copies have 93% and DRERV5 copies have 98%. The RT sequences from 1) these full length query DRERVs, 2) known fish retroviruses (endogenous and exogenous), and 3) representative retroviruses that the GPS pulls out of *O. latipes* and *G. aculeatus* were used to construct a phylogenetic tree (Figure 10). The DRERV clades have variable gene components. Using the methodology described above for the retrotransposon DRR1 LTRs, all but one of the DRERV families have LTRs, all with very high percent nucleotide identities. There are also 39 copies of DRERV4 in *G. aculeatus*, 12 of which are potentially active. The results of an in-depth study to further characterize these novel retroviruses in fish will be published shortly.

## Discussion

The purpose of this study is to create a global overview of Retroid agents in fish. Retroid agents comprise the largest class of transposable elements in Eukaryotes. Transposable elements can cause a range of effects on their host genomes including various types of mutations, which can modify the size and arrangement of an entire genome, and cause chromosomal rearrangements, including deletions, duplications, inversions, and reciprocal translocations. These rearrangements can cause genome reorganization, amplification, and reduction. Some transposable elements are suspected to preferentially insert into regions that do not contain host gene sequences in order to reduce their destructive influence on the host genome (Kidwell, 2002). There are numerous full length Retroid agents found by the GPS in the four fish genomes of this study, accompanied by an even larger number of RT gene fragments.

Unique, non-fish specific RT sequences not expected in fish, are nonetheless present in each of the four fish genomes. For example, there are small segments of RT sequences in the four fish that are more closely related to human RTs than they are to fish RT sequences. Non-fish specific RT queries (Figure 3) pull out large numbers of unique RT hits, which are generally smaller than the fish specific RT hits (Table 2), suggesting that these fragments are remnants of more ancient invasions. The fish genomes have a higher percentage of unique RT hits that are non-fish specific than the human genome has that are non-human specific (low frequency) (McClure et al. 2005). These non-fish specific/low frequency hits highlight the GPS’s ability to pull out very small and divergent remnants, and show that, although these small fragments are far from being active, their footprints are still present in the genome. New functionality will be added to the GPS to study what these remnants are, where they came from, and why they persist in all Eukaryotic genomes examined by the GPS to date.

All the fish specific queries (Figure 3) produced unique RT hits in all four fish genomes (data not shown). Not all fish genomes appear, however, to have full length or potentially active copies of the fish Retroid agents. In cases where Retroid agents are found in a subset of the four fish genomes (Figures 7 and 8, Table 3) little can be concluded as to the timing and mechanism (vertical or horizontal transfer) of insertion into a specific host until sequencing of the fish genomes is complete. The results presented here set the stage for the complete classification of all Retroid agents in fish genomes.

Six Retroid agents, however, BABAR, REX1, and REX3 (retroposons) and BARTHEZ2, RODIN and SUSHI-ICHI (retrotransposons) are found in all four fish analyzed (Figure 6). The presence of these Retroid agents in all four fish suggests that they inserted into a genome ancestral to the divergence of *D. rerio, O. latipes, G. aculeatus* and *T. nigroviridis*.

Although the count, type and distribution of Retroid agents will not be final until the genome sequencing and assembly is complete for all four fish genomes, some trends are clearly visible among and between these fish given the data generated by the GPS. Larger genomes are expected to have more transposable elements than smaller genomes. *D. rerio* with the largest genome of the four fish, has more total unique RT hits than *O. latipes*, *G. aculeatus* or *T. nigroviridis*. Looking at various classes of Retroid agents, however, suggests that not all agents follow the “more in larger genomes” idea. REX1 and ZEBULON each have more unique RTs and full length copies in *O. latipes*, a genome smaller than *D. rerio* (Figures 6 and 7). Perhaps these agents could not find an appropriate niche in *D. rerio* due to the large numbers of Retroid agents already residing there.

Interestingly, there is a disparity between the number of unique RTs versus full length and potentially active genomes for various Retroid agents. *D. rerio*, having the largest genome does indeed have more unique RT hits for almost all queries, but there are full length Retroid genomes that do not follow the expectation of more copies in the largest genome. The retroposons BABAR, REX3, KOSHITN1, MAUI, and TNDIRS1; the retrotransposons RODIN and SUSHI-ICHI and retroviruses ERV4_Tet and DRERV4 each have a higher full length copy number in a fish genome that is smaller than the largest one in which the agent is found (Figures 6, 7 and 8). For example, there are more RT signals for BABAR and REX3 in *D. rerio,* but there are more full length and potentially active copies in *O. latipes* (Figure 6). In most cases more full length Retroid genomes are correlated with more potentially active ones (Figures 6, 7 and 8). The fact that there is more unique RT signals in larger genomes, but fewer full length and potentially active copies suggests that there are many degraded copies in larger genomes. Using the GPS, an in-depth analysis of each query can be conducted to determine whether or not there are Retroid agents that have more than a RT gene, but less than a full length Retroid genome in larger host genomes. These data will reveal whether or not there are indeed degraded copies of these agents in larger genomes.

Those Retroid agents that are present in the most full length copies in larger genomes, like MUTSUDR3 and DRR1 (Figure 8), are expected to have similar selective pressures on them in both *D. rerio* and *O. latipes* (both of which are freshwater fish), allowing them to expand their host genomes. *D. rerio*, however, is much older that *O. latipes*, thereby having more time to accumulate these agents. One would expect that sequences like REX3 would have accumulated more copies in *D. rerio* than in the other three fish given its later divergence, but this is not the case (Figure 6). Retroid agents can be more degraded in one genome than in another due to relative insertion time, insertion site, and the selective pressure (or lack of selective pressure) on that section of the host’s genome. Only in a detailed analysis of each Retroid agent can its timing of insertion and subsequent fate be determined in an effort to understand the footprints that have been left in host genomes.

Transposable elements have been documented to insert into intergenic regions, heterochromatin, into or near other single copy sequences, or into other transposable elements (Kidwell, 2002). Because the GPS outputs the exact positions of each Retroid genome, the distribution of these sequences on chromosomes can quickly and easily be determined. We conducted a pilot study of full length Retroid genome distribution on chromosome one of all four fish. This chromosome was chosen because it does not have significantly higher or lower numbers of full length Retroid agents compared to the other chromosomes in all four fish. The GPS’s analysis of chromosome one of *T. nigroviridis* shows all full length copies fall on the second half of the chromosome, with no full length copies falling on the 5′ terminus of the chromosome. *O. latipes* has a very even distribution of full length sequences on its chromosome one, and in *D. rerio* only two out of 34 full length copies fall outside the middle two-thirds of the chromosome. *G. aculeatus*, however, has about 44% of its full length copies on chromosome one located in the 5′ and 3′ 10% of the chromosome. The GPS’s results for the current iterations of chromosome one sequences for the four fish indicate a variety of patterns of insertion for full length Retroid agents. Only the data from chromosome one of the *G. aculeatus* genome suggests some preferential insertion into heterochromatin over euchromatin as suggested by transposable element data in general. When taking into account all full length Retroid agents (including both retrotransposons and retroposons), the distribution of Retroid agents does not appear to show a preference for heterochromatin over euchromatin. A full study of Retroid agent chromosome position distribution cannot be conducted until all four fish genomes are completely sequenced and assembled.

The novel DRR1 agent is classified as a retrotransposon, rather than a retrovirus, because all of its gene components are closely related to other retrotransposon genes (data not shown), and there is no degraded ENV gene. Fourteen copies of the DRR1 retrotransposons have unique 5′ and 3′ LTRs that share a 69% nucleotide identity. These novel LTRs are unrelated to any LTR sequences from the NCBI viral and non-redundant databases as determined by a BLAST search.

The TERT sequences located in all four fish show the strength of the GPS method. Although the *D. rerio* genome sequencing is incomplete, the GPS was still able to detect the presence of a potential TERT gene, even though all six RT motifs are not present (Figure 9). Further assembly efforts will also place the *T. rubripes* sequence onto chromosomes, showing whether or not the TERT sequence is present on the same chromosome as the closely related pufferfish, *T. nigroviridis*. Synteny studies will also be of particular interest and will show whether or not the chromosome on which the TERT is located in one fish correlates to a similar location in the other fish genomes.

A number of novel retroviruses have been identified in *D. rerio*, *O. latipes* and *G. aculeatus*. Examples of these new retroviruses are shown in Figure 10, along with the known fish retroviruses. Phylogenetic reconstruction using RT amino acid sequences of all known fish retroviruses and the new ones discovered in this study suggest that there are three clades and two outliers of retroviruses (Figure 10). The first main clade includes the previously identified ERV3_Tet from *T. nigroviridis*, one lineage of novel *O. latipes* ERV3_Tet –like retroviruses, one of the new DRERV viruses (lineage four from *D. rerio*) and a lineage of new *G. aculeatus* DRERV4-like retroviruses. The second main clade includes the previously identified ERV2_Tet, the novel DRERV1 lineage from *D. rerio*, and the exogenous viruses walleye epidermal hyperplasia virus type 1 (WEHV1), walleye epidermal hyperplasia virus type 2 (WEHV2) and walleye dermal sarcoma virus (WDSV). The third clade includes ERV4_Tet, Atlantic salmon swim bladder sarcoma virus (SSSV), a novel retrovirus lineage from *O. latipes* the previously identified zebrafish endogenous retrovirus (ZFERV), the novel, single copy DRERV2 retrovirus from *D. rerio*, and a novel *G. aculeatus* retrovirus lineage. The novel DRERV3 and DRERV5 lineages are outliers and currently have no representatives in *O. latipes*, *G. aculeatus* and *T. nigroviridis* (Figure 10), but this may not be the case when sequencing is complete for these genomes. Low bootstrap values will be resolved when the more detailed analysis of fish retroviruses is complete. An in-depth study of fish retroviruses is in progress, and will include the phylogenetic reconstruction of each gene and the novel LTRs found in the results reported here.

The multiple families of retroposons found in all four fish are a contrast to the single family of retroposons found in primates. There are also a number of highly diverse, full length retrotransposons in the four teleost fish genomes, while human does not have any full length retrotransposons (McClure et al. 2005). There are many hypotheses on how and why diverse families of Retroid agents are maintained in compact genomes. Among eukaryotes, in general, larger genomes tend to have more transposable elements, of which Retroid agents are the largest subgroup, and it is proposed that these large genomes show a slower rate of deletion in both transposable elements and pseudogenes (Petrov, 2002). *T. nigroviridis* exhibits a more rapid deletion rate of repetitive pseudogenes than human does, which, combined with an apparent resistance to large insertions, may explain why *T. nigroviridis* has the smallest known vertebrate genome (Neafsey, 2003).

Further work on the Retroid agent content of fish will include the *Xiphophorus maculatus* (platyfish) and *T. rubripes* genomes when they are assembled into chromosomes. Comparing *T. rubripes* and *T. nigroviridis* will provide a look at how Retroid agents evolve over shorter divergence times in these two closely related pufferfish. Furthermore, when all sequences have been placed on chromosomes, a chromosomal position comparison can be conducted between the six fish species. This study will be of particular interest due to the whole genome duplication known to have occurred shortly after the teleost divergence (Jallion et al. 2004), illustrating which Retroid agents were present before this duplication and their behavior in subsequent speciation.

An extremely beneficial aspect of the GPS is the ability to analyze new releases of genomes efficiently and rapidly. We were able to add the *G. aculeatus* Retroid content to this paper within three days of the sequenced genome’s release. The GPS is able to find novel sequences, even when the LTRs are novel as well, as shown by the DRR1 and DRERV sequences. The GPS is also able to find novel TERT sequences, even when they are not entirely sequenced and have multiple introns. The GPS is a powerful method in identifying Retroid agents, with the capability of being applied to other elements in any genome and the ability to provide data for a very in-depth study of each element of interest as well as a global overview of Retroid agents across multiple genomes.

## Figures and Tables

**Table 1.** Stage I and Stage II GPS totals for *D. rerio*, *O. latipes***,** *G. aculeatus* and *T. nigroviridis.* Unique RT hits produced by Stage I GPS are indicated for each of the four fish in the first column, and the full length Retroid agents produced by Stage II GPS are shown for each fish in the second column.

**Organism**	**Unique RT Hits**	**Full Length Retroid Agents**
*D. rerio*	102,763	1,116
*O. latipes*	61,767	1,458
*G. aculeatus*	31,865	777
*T. nigroviridis*	18,322	633

**Table 2.** Average Sizes of Fish Specific and Non-fish Specific Unique RT Hits from Stage I GPS. All values are indicated in base pairs (bp).

**Organism**	**Fish Specific Hits**	**Non-fish Specific Hits**
*D. rerio*	243 bp	163 bp
*O. latipes*	221 bp	166 bp
*G. aculeatus*	222 bp	144 bp
*T. nigroviridis*	161 bp	120 bp

**Table 3.** Stage II GPS results: full length and potentially active Retroid agents specific to each fish. **A)** Retroid agents found in full length only in the *D. rerio* genome. **B)** Retroid agents found in full length only in the *O. latipes* genome. **C)** Retroid agents found in full length only in the *T. nigroviridis* genome. There are no Retroid agents found solely in the *G. aculeatus* genome. Retroid agents are grouped into retroposon, retrotransposon and retrovirus families, and their hosts of origin and accession numbers are shown in Figure 3.

	**Full**	**Potentially Active**
**A)*D. rerio***		
**Retroposons**		
DEWADR1	24	5
KIBIDR1	5	0
KIBIDR2	14	1
R2DR	2	2
**Retrotransposons**		
DREGG1	141	53
**Retroviruses**		
DRERV1	11	4
DRERV2	1	0
DRERV3	6	3
DRERV5	7	1
ZFERV	4	0
**B)*O. latipes***		
**Retrotransposons**		
REX8	1	0
**C)*T. nigroviridis***		
**Retrovirus**		
ERV2_Tet	2	0

**Table 4.** Location of Fish TERTs and their Percent Identity to the FTERT Query. The first column is organismal TERT gene name, the second is the chromosome on which each TERT is located and the third is the percent identity of each new TERT to the FTERT query.

**Agent**	**Chromosome**	**%aa id to FTERT**
DRTERT	19	43%
OLTERT	11	68%
GATERT	10	73%
TNTERT	21	80%
